# Exogenous Probiotics Improve Fermentation Quality, Microflora Phenotypes, and Trophic Modes of Fermented Vegetable Waste for Animal Feed

**DOI:** 10.3390/microorganisms9030644

**Published:** 2021-03-19

**Authors:** Guilin Du, Jiping Shi, Jingxian Zhang, Zhiguo Ma, Xiangcen Liu, Chenyang Yuan, Baoguo Zhang, Zhanying Zhang, Mark D. Harrison

**Affiliations:** 1 Lab of Biorefinery, Shanghai Advanced Research Institute, Chinese Academy of Sciences, No. 99 Haike Road, Pudong, Shanghai 201210, China; dugl1@shanghaitech.edu.cn (G.D.); shijp@sari.ac.cn (J.S.); zhangjingxian@sari.ac.cn (J.Z.); mazhiguo2018@sari.ac.cn (Z.M.); liuxiangcen2019@sari.ac.cn (X.L.); yuanchy@shanghaitech.edu.cn (C.Y.); 2 University of Chinese Academy of Sciences, Beijing 100049, China; 3 School of Life Science and Technology, ShanghaiTech University, Shanghai 201210, China; 4 Centre for Agriculture and the Bioeconomy, Faculty of Science, Queensland University of Technology, Brisbane, QLD 4000, Australia; jan.zhang@qut.edu.au (Z.Z.); md.harrison@qut.edu.au (M.D.H.); 5 School of Mechanical, Medical and Process Engineering, Faculty of Engineering, Queensland University of Technology, Brisbane, QLD 4000, Australia; 6 School of Biology and Environmental Science, Faculty of Science, Queensland University of Technology, Brisbane, QLD 4000, Australia

**Keywords:** vegetable waste, fermented feed, microbial diversity, bacterial phenotypes, fungal functional guild, network analysis

## Abstract

The fermentation of leaf vegetable waste to produce animal feed reduces the environmental impact of vegetable production and transforms leaf vegetable waste into a commodity. We investigated the effect of exogenous probiotics and lignocellulose enzymes on the quality and microbial community of fermented feed (FF) produced from cabbage waste. The addition of exogenous probiotics resulted in increased crude protein (CP) content (*p* < 0.05), better odor (moderate organic acid and ethanol, with low ammonia-N, *p* < 0.05), and a lower relative abundance (RA) of pathogens (below 0.4%, *p* < 0.05) in FF, compared to without. With the addition of exogenous probiotics, only *Pediococcus* and *Saccharomyces* were enriched and symbiotic in FF; these were the keystone taxa to reduce the abundance of aerobic, form-biofilms, and pathogenic microorganisms, resulting in an efficient anaerobic fermentation system characterized by facultative anaerobic and Gram-positive bacterial communities, and undefined saprotroph fungal communities. Thus, inoculation of vegetable waste fermentation with exogenous probiotics is a promising strategy to enhance the biotransformation of vegetable waste into animal feed.

## 1. Introduction

Improved standards of living result in changes in the way in which horticultural products are selected for harvesting, storage, transport, and sale. This is particularly true of leafy vegetables such as cauliflower, white cabbage, leek, and carrots [1,2,3]. Vegetable cultivation accounts for more than 10% of major crop cultivation in China and is critical in meeting human nutritional requirements, but it also generates 800 million tons of vegetable waste per annum. This vegetable waste causes environmental challenges including plant/animal pathogen propagation, atmospheric and water pollution, and greenhouse gas emissions [3,4,5,6]. Vegetable wastes are typically seasonal and have high (80 wt.%) moisture content, abundant macro- and micro-nutrients, and a relatively high pathogen load [1,7]. Several strategies for vegetable waste management are available; for example, composting and anaerobic co-digestion can convert vegetable waste into eco-friendly fertilizer and biogas, respectively [1,7,8]. However, relative to the production of fertilizer and energy, it is simpler and more economical to treat vegetable waste as a source of animal feed because it preserves the nutritional value and transforms vegetable waste into (ultimately) relatively high-value animal feed [7,9,10]. Therefore, research describing the transformation of vegetable waste into animal feed has national and international significance in the face of increasing environmental pollution and the role of animal production in food security.

The transformation of waste from human food production (such as agricultural by-products and food preparation wastes) into novel animal feeds has been widely reported [9,11,12,13,14,15]. However, there are significant constraints in the use of these raw materials in animal feed production because of their putrescible characteristic and the presence of endogenous antinutritional factors, pathogens, and mycotoxins [3,12,16]. Production of FF from human food production wastes has attracted significant interest because microbial fermentation can be undertaken using a wide range of substrates, preserves the nutrients, degrades antinutritional factors, and limits the growth of pathogens and mycotoxin content [17]. The addition of exogenous probiotics and, therefore, the extracellular enzymes they secrete, can enhance the production of FF by preventing decomposition, degrading macronutrients, improving overall nutritional value, improving sensory characteristics, and exerting a positive effect on the microbiome of the animal gastrointestinal tract that enhances animal health and productivity [3,17,18,19]. For example, the application of *Lactobacillus plantarum* to the fermentation of cauliflower leaf waste increased the RA of lactic acid bacteria [20], thereby resulting in more efficient lactic acid fermentation, reduced proteolysis, and reduced dry matter (DM) loss [3]. The inoculation of more nutritious feedstuffs, such as corn, soybean meal, and distiller’s grains with probiotics such as *Bacillus subtilis*, *L. plantarum*, and *Saccharomyces cerevisiae* also enhances their nutritional value in animal feed by increasing CP content, reducing the average protein molecular mass, decreasing the content of antinutritional compounds (such as soybean antigenic proteins glycinin and β-conglycinin), and decreasing lignocellulose content [12,17,21,22]. Furthermore, the addition of lignocellulose enzymes, such as cellulase and xylanase, alone or in combination with LAB, to the fermentation of food waste for animal feed can enhance the hydrolysis of structural carbohydrates to water-soluble sugars and their subsequent microbial transformation into organic acids [23,24]. The combination of next-generation sequencing (NGS) and bioinformatics systems, such as BugBase and FUNGuild, has been used to investigate the composition and characteristics of complex microbial communities associated with environmental samples [23,25,26,27]. However, there are only a limited number of studies focused on the microbial community dynamics, microorganic phenotypes, and trophic modes in FF.

In the present study, vegetable wastes were mixed with other non-competing human food waste to produce nutritious, low-cost fermented animal feed. The two main objectives of this study were to (i) determine whether fermentation quality, microbiome structure, bacterial community phenotypes, and fungal community trophic modes changed in response to the addition of exogenous probiotics and enzyme additives; and (ii) identify which microbial taxa could influence the aforementioned indicators in vegetable waste FF. To our best knowledge, this study is the first time that the microorganic phenotype, trophic mode, and RA of potential pathogens have been evaluated in FF. The results of the study highlight the potential value of fermentation as a viable option to reduce the environmental impact of vegetable waste, the potential of FF as an animal feed, the key microbial taxa needed for the production of high-quality FF, and their function during the fermentation of vegetable waste.

## 2. Materials and Methods

### 2.1. Preparation of Materials and Additives

Wheat (*Triticum aestivum* L.) bran, soybean (*Glycine max* (Linn.) Merr.) meal, and corn (*Zea mays* L.) flour were purchased from Beicai Agricultural Products Wholesale Market (Shanghai, China). Cabbage (*Brassica oleracea* L.) leaf waste was collected from the same market. Cabbage leaf waste was shredded to a width of 1–2 cm; the remaining raw feed ingredients did not require further processing. The chemical composition of the raw feed ingredients are presented in Table 1, while detection methods are described in Section 2.4. Exogenous probiotics (*S. cerevisiae* (CICC NO.1421), *B. subtilis* (CICC NO. 20872), and *L. plantarum* (CGMCC NO.19862)) were provided by Henan Xinyangshao Bio-Technology Co., Ltd. (Sanmenxia, China) The enzyme mixture containing xylanase, cellulase, and β-glucosidase (Item No. SFG-0950) was purchased from Sunson Industry Group Co., Ltd. (Cangzhou, China).

### 2.2. Fermented Feed Set-Up and Sampling

The experiment consisted of three treatment groups: a control group with no added probiotics or enzymes (CTGP), a probiotics treatment group (PTGP), and an enzyme treatment group (ETGP). The formulations of the three fermentation treatment groups are presented in Table 2, and sufficient sterile water was added to achieve 62% moisture content. The pH of the three treatment groups was not adjusted. Feed formulations (~500 g) were placed into PET plastic bags (23 cm × 30 cm; Hongxu plastic bag Co., Ltd., Wenzhou, China) and sealed with a vacuum sealer (Blueberry 320X, Shanghai Inuo Packaging Materials Co., Ltd., Shanghai, China). A total of 15 bags per treatment group were fermented in an incubator at 30 °C for 15 days. Three bags from each treatment group were removed at days 0, 2, 5, 10, and 15, and sub-sampled for analysis.

### 2.3. Analytical Produce

The first sub-samples (~20 g) were mixed with 180 mL of sterile 0.8% (*w*/*v*) NaCl (Sinopharm, Shanghai, China) solution in a 500 mL conical flask and agitated at 30 °C for 2 h using a rotary shaker at 150× rpm. The mixed solution was filtered through four layers of medical gauze. The mixture of relatively small substrate particles and microbial biomass was collected by centrifugation at 4 °C for 20 min at 10,000× *g*. The pellets from each of the three replicates’ treatment groups were stored at −80 °C until required for microbiome analysis. The other sets of extracts were prepared from the second sub-samples of each treatment group using sterile water and the method described above. The supernatants from these extracts were used to measure physicochemical indicators [28].

### 2.4. Analyses of the Physicochemical Properties

The physicochemical indicators that were evaluated were as follows: pH, DM, CP, crude fat (CF), neutral detergent fiber (NDF), acid detergent fiber (ADF), total nitrogen (TN), ammonia-N, organic acid (lactic acid, formic acid, acetic acid, propionic acid, butyric acid), and ethanol contents. The filtered supernatant (described in Section 2.2) was used to measure the organic acid and ethanol contents using a Shimadzu 20AVP liquid chromatography system (HPLC) (Shimadzu Corp., Kyoto, Japan) equipped with a RID-10A refractive index detector and a SPD-M20A photodiode array detector. An AminexHPX-87H column (300 × 7.8 mm) (Bio-Rad, Hercules, CA, USA) was used at the column temperature, 65 °C; a 0.005 mol/L H_2_SO_4_ solution was used as the mobile phase at a velocity of 0.8 mL/min. Retention time of the sample was 20 min, while peak times of lactic acid, formic acid, acetic acid, propionic acid, butyric acid, and ethanol were 9.9 min, 10.3 min, 11.3 min, 13.0 min, 14.7 min, and 16.2 min, respectively. The standard reagents of organic acids and ethanol were obtained from Sigma-Aldrich Co., Ltd. (USA). Ammonia-N was quantified using Nessler’s reagent (Hach, USA), and pH was determined using a digital pH meter (PB-10, Sartorius, USA), as per the manufacturer’s instructions. FF (~50 g) was dried at 65 °C for 72 h to determine the moisture content [29]. The dry sample was ground and sieved through a 1 mm screen, and used for subsequent analyses. The content of CP and TN were determined using a Kjeldahl nitrogen analyzer. CF content was determined via Soxhlet extraction (B-811, BUCHI, Switzerland), and the defatted sample was subsequently used to measure ADF and NDF content using a Fibretherm (C. Gerhardt, Germany), as per the manufacturer’s instructions. The equality of FF was evaluated by Flieg’s score based on the percent of lactic acid, acetic acid, and butyric acid in organic acid [30].

### 2.5. DNA Extraction and MiSeq Sequencing

The 0.25 g pellets described in Section 2.2 were used to extract total microbial DNA using an E.Z.N.A.^®^ soil DNA Kit (Omega Bio-Tek, Norcross, GA, USA), as per the manufacturer’s instructions. DNA concentration and purity were measured using a NanoDrop 2000 UV–vis spectrophotometer (Thermo Scientific, Wilmington, NC, USA). All DNA samples were stored at −80 °C until required. PCR amplification of the 16S rDNA and internal transcribed spacer (ITS) regions was performed as described previously [31]. The V3–V4 hypervariable regions from prokaryotic 16S rRNA and the ITS region from eukaryotic ITS rRNA were amplified using the barcoded fusion primers 338F (ACTCCTACGGGAGGCAGCAG), 806R (GGACTACHVGGGTWTCTAAT), ITS1F (CTTGGTCATTTAGAGGAAGTAA), and ITS2R (CTTGGTCATTTAGAGGAAGTAA), respectively. DNA quality was confirmed by agarose gel electrophoresis. PCR products were sent to Majorbio Bio-pharm Technology Co., Ltd. (Shanghai, China) for further purification, extraction, and sequencing, as described previously [32].

### 2.6. Bioinformatics Analyses

The raw Illumina fastq files were demultiplexed, quality filtered, and analyzed using QIIME (v. 1.9.1 http://qiime.org/, accessed on 30 December 2019). Alpha diversity, including Shannon, Chao1, and coverage indexes of every sample was calculated with MOTHUR software (v. 1.30.2; https://www.mothur.org/, accessed on 30 December 2019). Subsequently, the quality-filtered sequences were clustered into operational taxonomic units (OTUs) with a 97% similarity cut-off using UPARSE (v. 7.1 http://drive5.com/uparse/, accessed on 30 December 2019), and chimeric sequences were removed using UCHIME algorithm (http://www.drive5.com/uchime/, accessed on 30 December 2019). The taxonomy of each 16S and ITS rDNA sequence was annotated by alignment with Silva 132 (https://Awww.arb-silva.de/, accessed on 30 December 2019) and Unite 7.2 (https://unite.ut.ee/, accessed on 30 December 2019) using the RDP Classifier (v. 2.2; http://sourceforge.net/projects/rdp-classifier/, accessed on 30 December 2019) with a confidence threshold of 70%. The bacterial phenotypes were predicted and analyzed using BugBase (https://bugbase.cs.umn.edu/, accessed on 30 December 2019) [27]. Fungal trophic modes and ecological guilds were analyzed using FUNGuild (http://www.funguild.org/, accessed on 30 December 2019) [26]. All analyses of MiSeq sequencing were performed using the free online platform provided by the Majorbio I-Sanger Cloud Platform (http://www.i-sanger.com/, accessed on 30 December 2019). The dissimilarity of microbial communities among different samples was calculated via principal coordinate analysis (PCoA) on the level of OTUs. The significantly different (*p* < 0.05) taxa during anaerobic fermentation in different groups were identified using linear discriminant analysis coupled with effect size measurement (LEfSe) analysis (LDA score > 4.0) [33]. The effect of fermentation additives on the microflora succession was assessed using the Adonis test on the OTU level (999 random permutations, Bray–Curtis dissimilarity).

### 2.7. Correlation between Microbial Genus and Physicochemical Indexes

Correlations (linear and simple nonlinear) among different variables were evaluated using Spearman’s rank correlation analysis. Only genera of RA > 1% were considered. To highlight the most important interrelationships, a correlation network was used to visualize the strong correlations (Spearman coefficient > 0.8 or <−0.8, *p* < 0.05) with Cytoscape 3.8.0 [32,34]. Network topology properties were analyzed with the NetworkAnalyzer tool. The Network Randomizer 1.1.3 plugin was used to assess non-random patterns by comparing our networks against the most similar networks that were randomly generated with the same node and degree value. Topological attributes, such as clustering coefficient, indicated that the shortest path and network diameter and radius were significantly different between the randomly generated networks and our experimental network. MCODE 1.6.1 was used to analyze densely connected regions with standard parameters as previously described [32,35].

### 2.8. Statistical Analysis

Physicochemical indexes were analyzed using two-way ANOVA for a 3 × 5 (3 treatments × 5 sampling times) full factorial experimental design with three replicates (IBM SPSS 26.0, New York City, NY, USA). Significant differences between each treatment were determined by the Tukey test (α = 0.05 and *P_critical_* = 0.05). Principal component analysis (PCA) application was used to evaluate the relationships among the physicochemical indexes during anaerobic fermentation (OriginPro 2020b, Northampton, MA, USA).

## 3. Results and Discussion

### 3.1. Effects on the Physicochemical Properties

The results of analysis of the physicochemical properties of FF after 0–15 days of anaerobic fermentation are presented in Table 3, Table 4 and Table 5. Physical and chemical indicators were significantly influenced by the exogenous additive and fermentation time in this study. CP and CF content in PTGP was 29.5% higher on day 5 and 76.7% higher on day 15, respectively, when compared to CTGP. Ethanol content in PTGP was also increased on day 5 (66.3 g kg^−1^ DM) compared to CTGP. The lactic acid content in PTGP increased to 63.4 g kg^−1^ DM on day 5, but was significantly lower than that observed for CTGP and ETGP, which may have been due to substrate competition between ethanol-producing yeasts and LABs or metabolism of lactate by exogenous probiotic *S. cerevisiae*. Furthermore, the addition of exogenous probiotics resulted in higher pH and better sensory qualities for PTGP compared to CTGP and ETGP. Enhanced sensory quality in PTGP was likely the result of moderate organic acid and ethanol content. Ammonia-N content in all FFs were less than 5%, which is appropriate in silage [36]. Interestingly, the ammonia-N content of PTGP was significantly lower after day 10 compared to CTGP and ETGP. Ammonia-N has been reported as a product of deamination activity of proteins, peptides, and amino acids by undesirable microbes during ensiling, which is an indicator of nutrition loss [3,36]. Thus, the probiotics and their products (such as ethanol and organic acid) limited the growth of undesirable microflora and likely reduced proteolytic activity. Similar results have been reported on silage by He et al. [36] and Ren et al. [3].

Moreover, the organic acid ingredient is regarded as an important indicator to evaluate the silage odor, and Flieg’s score > 81 indicates very good quality [30]. In this study, all treatment groups were evaluated with “very good” after day 5 (Table 5). However, the sweet-scented odor of fermented feed also depended on other physicochemical indexes, such as more ethanol and lower ammonia-N content. Actually, FFs with the exogenous probiotics presented the best odor due to moderate organic acid ingredients, ethanol, and ammonia-N content. Therefore, exogenous probiotics could be a feasible method to transform cabbage waste into fermented animal feed.

**Figure 1:**
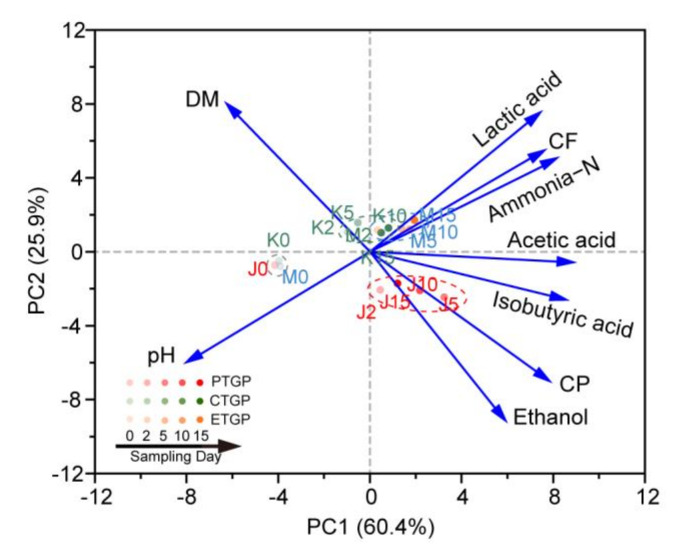
Principal component analysis of samples and major physicochemical indicators. DM: dry matter content; CP: crude protein content; CF: crude fat content. PTGP: the probiotics treatment group; CTGP: the control group; ETGP: the enzyme treatment group. The numbers following the J/K/M indicate the sampling time (day) of PTGP/CTGP/ETGP.

To determine the relationships between the physicochemical indicators of anaerobic fermentation, principal component analysis (PCA) was performed (Figure 1). The first two axes of PCA accounted for 86.3% of the total variance; ethanol and CP content had a major influence on the physicochemical properties of PTGP, whereas lactic acid, CF, and ammonia content had a major influence on those properties in CTGP and EPGP. Additionally, lactic acid content was positively correlated with short-chain fatty acids, CF, and ammonia content, but negatively correlated with pH. This indicated that the bacteria producing lactic acid, such as *Pediococcus acidilactici*, could also produce other short-chain fatty acids, which decreased pH. Interestingly, LAB may have also promoted the accumulation of CF, particularly in the ETGP group (Table 3). Similarly, the ethanol content was positively correlated with CP content and negatively correlated with DM according to the fungal metabolism reaction, which was noticeable in PTGP.

### 3.2. Effects on the Microflora Diversity and Richness

DNA sequencing coverage of each sample was above 0.978 (Table S1), which indicated that the refined OTUs were representative of the microbial communities thereon. Shannon and Chao1 indexes indicate microbial diversity and richness, respectively, which decreased in all samples due to the development of an acidic, anaerobic environment during fermentation. Interestingly, diversity and richness were significantly lower in the PTGP group during fermentation than either CTGP or ETGP (*p* < 0.05). These results suggest that exogenous probiotics and abundant ethanol decreased the colonization of undesirable microorganisms in PTGP, which resulted in the reduced richness and diversity compared with CTGP and ETGP observed in this study. These results were similar to those obtained by Mu et al. [23] after inoculation of amaranth and rice straw silage with *L. plantarum*, which led to declining richness and diversity.

### 3.3. Effects on the Microflora Succession

**Figure 2:**
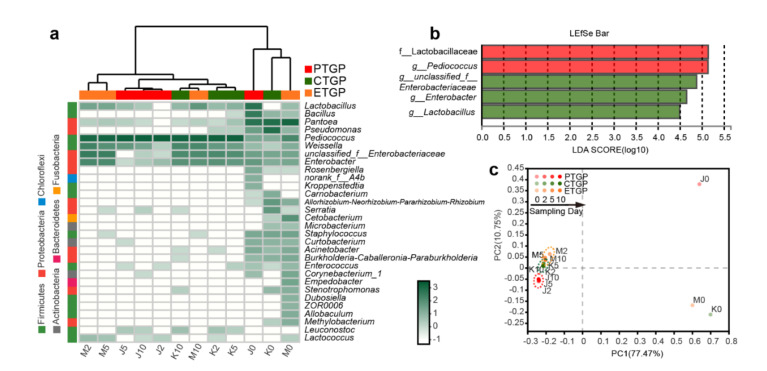
The bacterial community succession was associated with exogenous probiotics and enzymes during fermentation. (**a**) Heat maps showing the relative abundance of the top 30 bacterial genera in the fermented feed; the color intensity presents the log-transformed value of standardized reads. (**b**) Linear discriminant analysis (LDA) coupled with the effect size measurements identifies the significant abundance of data in (**a**); LDA significant threshold was >4.0. (**c**) Principal coordinate analysis based on bacterial OTU levels. The numbers following the J/K/M indicate the sampling time (day) of PTGP/CTGP/ETGP.

**Figure 3:**
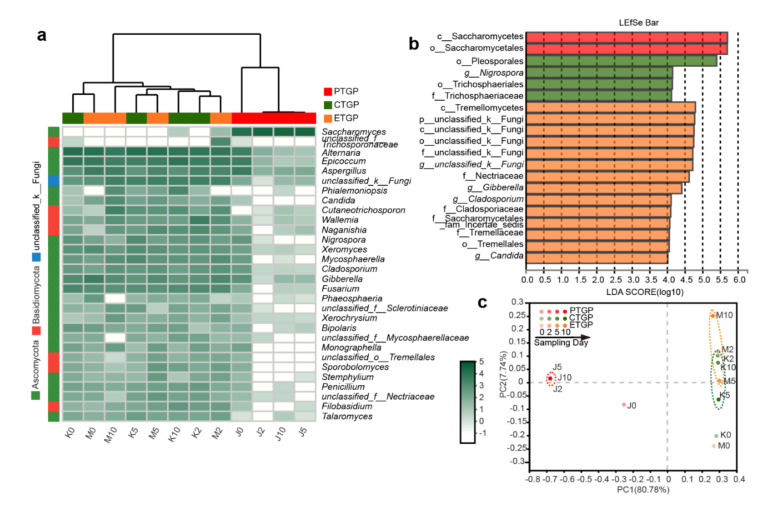
The fungal community succession was associated with exogenous probiotics and enzymes during fermentation. (**a**) Heat maps showing the relative abundance of the top 30 fungal genera in the fermented feed; the color intensity presents the log-transformed value of standardized reads. (**b**) Linear discriminant analysis (LDA) coupled with the effect size measurements identifies the significant abundance of data in (**a**); LDA significant threshold was >4.0. (**c**) Principal coordinate analysis based on fungal OTU levels. The numbers following the J/K/M indicate the sampling time (day) of PTGP/CTGP/ETGP.

Dynamic changes in the bacterial and fungal communities at the genus level were observed during anaerobic fermentation of cabbage waste (Figure 2a and Figure 3a). PCoA clearly showed dynamic succession of microflora in all three treatment groups (Figure 2c and Figure 3c). Microflora succession in the fermentation feed was significantly influenced by the presence of exogenous probiotics and enzymes after day 0 (Adonis test, *p* < 0.05). The mixed raw materials were enriched with Proteobacteria (*Pantoea*, *Pseudomonas*) and Ascomycota (*Alternaria*, *Epicoccum*, *Fusarium*), both of which are common endogenous bacteria and fungi, respectively, in silage and other environmental samples [3,16,18,31,37]. However, the above genera (except for *Pantoea*) are undesirable and potentially pathogenic in feed [16]. Interestingly, probiotic inoculation in PTGP reduced the abundance of the aforementioned genera during anaerobic fermentation. Additionally, among the exogenous compounded probiotics, only fungal *Saccharomyces* became the dominant taxon (average RA, 99.1%). Meanwhile, endogenous bacterial *Pediococcus* and *Weissella* predominated in the PTGP group (average RA, 97.7%, and 1.5%, respectively), which were the prevalent LAB in silage [3,28]. The dominant genera and OTUs in PTGP did not change significantly after day 3 (Figure 2c and Figure 3c), which indicated favorable probiotic microflora in the FF. The microflora succession and dominant genera in CTGP and ETGP during anaerobic fermentation were similar relative those in PTGP. LAB, including endogenous *Pediococcus* and *Weissella* and exogenous *Lactobacillus*, accounted for 88.5% in CTGP and 80.0% in ETGP, respectively. However, endogenous and undesired *unclassified_f__Enterobacteriaceae*, *Enterobacter* were preserved during the entire fermentation process, accounting for 11.0% and 19.6% in the ETGP and CTGP, respectively. *Unclassified_f__Enterobacteriaceae* and *Enterobacter* have been reported as undesirable bacteria in silage because they exhibit proteolytic activity while enriching ammonia-N [36]. This was consistent with the reduced CP and increased ammonia-N in CTGP and ETGP compared to PTGP. Similar results were observed for the fungi at the genus level. Endogenous and undesired *Alternaria*, *Epicoccum*, *Aspergillus*, *unclassified_k__Fungi*, *Gibberella*, and *Cutaneotrichosporon* dominated during anaerobic fermentation, accounting for 76.6% and 75.9% of the fungal community in CTGP and ETGP, respectively. These fungal genera have been reported as typical pathogens of plants and/or animals and can produce multiple mycotoxins in feed matrices [16,38,39]. Thus, the application of exogenous probiotics was necessary and efficient on amendment of pathogenic bacterial and fungal community, which was consistent with what Ren et al. [3] and Yang et al. [40] reported in silage with *L. plantarum*.

LEfSe analysis was performed to compare the microorganic variations from the phyla to genus level in FF (Figure 2b and Figure 3b). During the fermentation, bacterial *f__Lactobacillaceae* and *g__Pediococcus* and fungal *o__Saccharomycetales* and *c__Saccharomycetales* were only enriched in PTGP. In CTGP, the RA of fungal *o__Trichosphaeriales*, *f__Trichosphaeriaceae*, and *g__Nigrospora* were higher than was observed in the PTGP and ETGP groups. Interestingly, the addition of exogenous enzymes enabled unique bacterial (*g__Lactobacillus*, *g__Enterobacter*, *g__unclassified__Enterobacteriaceae*) and fungal (*p__unclassified__k__Fungi*, *c__Tremellomycetes*, and *f__Nectriaceae*) taxa to reach the threshold for detection. Thus, exogenous probiotics and enzymes influenced microflora succession in PTGP and ETGP, and the addition of exogenous probiotics was an effective method to improve the overall quality of the microbiota in cabbage leaf FF.

### 3.4. Effects on the Microflora Characteristics

#### 3.4.1. Effects on the Phenotype of Bacterial Communities

**Figure 4:**
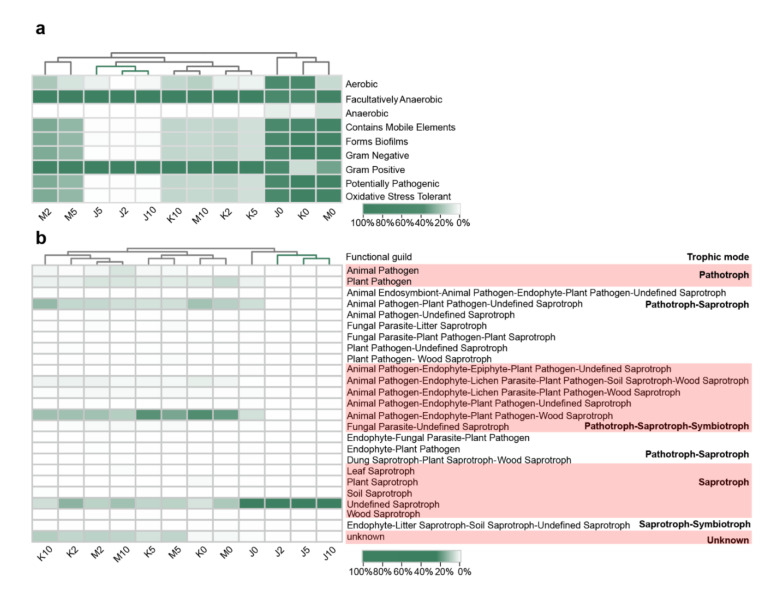
Heat maps showing the dynamics of microflora characteristics of the samples with different additives in the fermented feed. (**a**) The bacterial community phenotypes based on BugBase. (**b**) Fungal community trophic mode and functional guild based on FUNGuild. Only the top 25 guilds are shown in the heat map. The numbers following the J/K/M indicate the sampling time (day) of PTGP/CTGP/ETGP.

**Figure 5:**
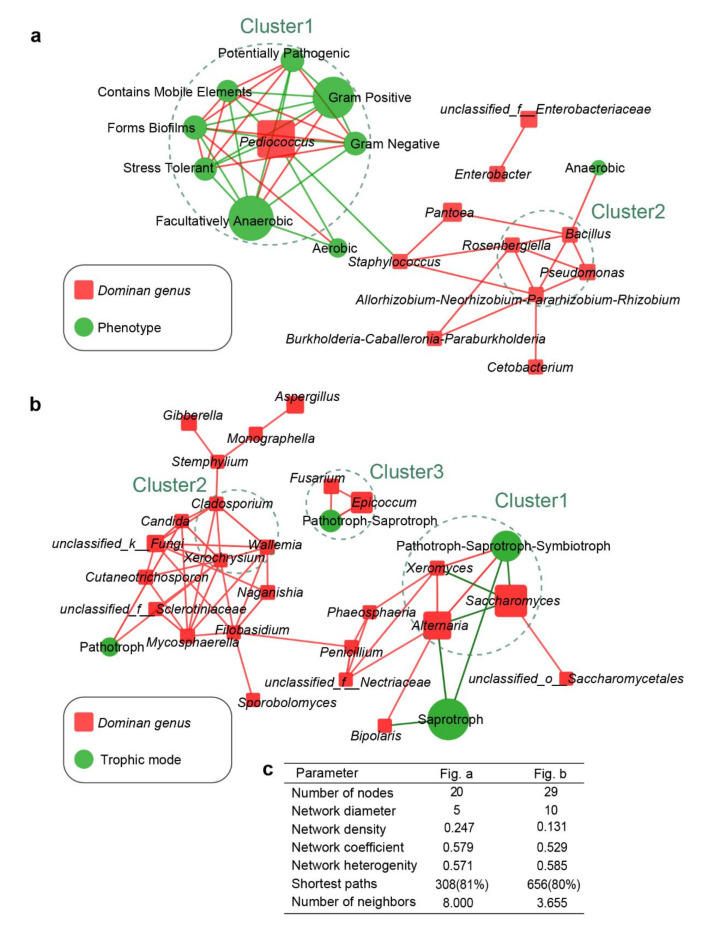
Network analysis based on the correlation between microflora and microflora characteristics. (**a**) Bacterial community phenotypes and bacterial genera. (**b**) Fungal community trophic mode and fungal genera. Red edges: positive correlation; green edges: negative correlation; the size of nodes indicates the average relative abundance of the genera/phenotypes/trophic modes. (**c**) The topological parameters of networks.

Phenotypic characteristics of the bacterial communities in FF were initially analyzed with BugBase to determine bacterial function. The results predicted nine potential phenotypes, including those that were potentially pathogenic, aerobic, anaerobic, facultatively anaerobic, contained mobile elements, biofilm-forming, Gram negative, Gram positive, and oxidative-stress tolerant (Figure 4a). Under an anaerobic environment, the RAs of bacterial communities that were aerobic, anaerobic, and oxidative-stress-tolerant decreased, whereas facultatively anaerobic bacterial communities were dominant. Bacteria that were Gram negative, potentially pathogenic, containing mobile elements and formed biofilms also declined. Interestingly, the presence of probiotics enhanced these tendencies, which resulted in distinct differences between PTGP and the other two FF types (CTGP, ETGP) in hierarchical clustering based on bacterial phenotypes. Fewer potentially pathogenic (average RA of 0.28%), form-biofilms (average RA of 1.2%), and aerobic bacteria (average RA of 1.6%) existed in PTGP during anaerobic fermentation compared with CTGP and ETGP. As previously reported, abundant aerobic bacteria enriched in silage could lead to aerobic deterioration and poor nutrition preservation [28,41]. Furthermore, biofilms-forming bacteria could tightly attach materials and resist antibiotics and high-temperature pressure to result in a series of health problems [42,43]. Therefore, with the exogenous probiotic inoculation, bacterial community was efficiently improved by declining the RA of undesirable bacteria. These results could be explained by keystone and dominant bacteria (endogenous *Pediococcus*) playing critical roles in the change of bacterial phenotypes. This hypothesis was consistent with the result that *Pediococcus* was closely correlated to bacterial community phenotypes in the correlation network among bacterial genera and bacterial phenotypes (Figure 5a). Therefore, endogenous *Pediococcus* was the keystone taxon that influenced the bacterial community phenotypes in the present study.

#### 3.4.2. Effects on the Trophic Modes of the Fungal Communities

Fungal trophic modes and ecological guilds were annotated using FUNGuild based on the OTU level (Figure 4b). Fungal trophic modes and guilds showed significant variation during anaerobic fermentation. This clear demarcation between FF types allowed us to separate all samples into two major hierarchical clusters based on fungal ecological guilds. The first cluster consisted of all the PTGP samples, and the second cluster consisted of all CTGP and ETGP samples. Fungal trophic modes and guilds mainly included pathotrophs (plant pathogen, 8.3%), pathotrophs–saprotrophs (animal pathogen–plant pathogen–undefined saprotroph, 23.6%), pathotrophs–saprotrophs–symbiotrophs (animal pathogen–endophyte–plant pathogen–wood saprotrophs, 44.5%), and saprotrophs (undefined saprotrophs, 10.1%) in the raw materials. During anaerobic fermentation, only saprotroph (undefined saprotroph) fungi primarily remained in PTGP, and pathotrophs–saprotrophs (animal pathogen–plant pathogen–undefined saprotrophs), pathotrophs–saprotrophs–symbiotrophs (animal pathogen–endophyte–plant pathogen–wood saprotrophs), saprotrophs (undefined saprotrophs), and unknown fungi were dominant in the CTGP and ETGP. The RAs of pathotroph and the other compatible pathotroph modes were significantly lower in PTGP compared to CTGP and ETGP. This was particularly apparent after two days of anaerobic fermentation. The RA of pathotrophic fungi decreased to less than 0.4% in PTGP, whereas the RAs of the plant pathogen, animal pathogen, fungal parasite, and unannotated guilds were relatively high (80.3% and 79.8%, respectively) in CTGP and ETGP. Therefore, the exogenous probiotics additive (especially *S. cerevisiae*) efficiently eliminated pathotrophic fungi during the anaerobic fermentation of cabbage waste FF. Given the RA of fungal community, exogenous *Saccharomyces* could be the keystone taxa that influence the fungal trophic modes and functional guilds in PTGP, which were consistent with the results that *Saccharomyces* negatively correlated with pathogenic fungi and trophic modes in the correlation network among fungal genera and trophic modes (Figure 5b).

### 3.5. Relationships between Microflora and Their Physicochemical Characteristics

**Figure 6:**
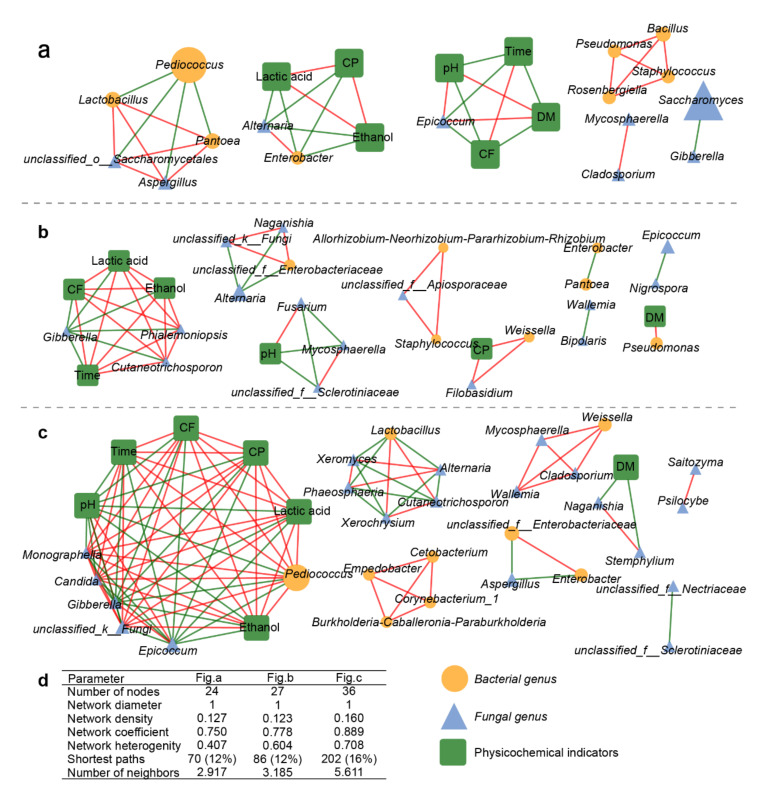
Network analysis showing co-occurrence and modular patterns based on the correlation among microbial communities and physicochemical indicators in response to exogenous probiotics (**a**), no treatment (**b**), and exogenous enzymes (**c**). Red edges: positive correlation; green edges: negative correlation; the size of nodes indicates the average relative abundance of the genera. (**d**) The topological parameters of networks. Time: fermented time; DM: dry matter; CF: crude fat; CP: crude protein.

Correlation networks among the dominant bacterial and fungal genera and the physicochemical characteristics for the different treatment groups were constructed to understand the effects of different additives on the correlation between fermentation quality and microflora structure (Figure 6). Of note was the alteration of co-occurrence patterns and network modularity in PTGP and ETGP relative to CTGP. The correlation network in CTGP comprised 27 nodes and the average number, coefficient, heterogeneity, shortest path length, and average neighbor number were 0.778, 0.604, 86, and 3.185, respectively (Figure 6a). In CTGP, *Alternaria* (average RA > 10%) was a common plant and animal pathogen [16,44], which correlated negatively with *unclassified_f__Enterobacteriaceae*, *unclassified_k__Fungi*, and *Naganishia*. The addition of probiotics in PTGP decreased the number of nodes (24), coefficient (0.750), heterogeneity (0.407), shortest path length (70), and average neighbor number (2.917) of the correlation network (Figure 6b), which indicated few interactions in PTGP. These results demonstrated that abundant exogenous probiotics directly changed the initial microflora structure so that subsequent microbial diversity and interaction in PTGP was lower than in CTGP. Moreover, with the addition of the probiotics, endogenous *Pediococcus* was the keystone taxon (average RA > 10%) and negatively correlated with *Pantoea, Aspergillus*, and *Lactobacillus* (Figure 6a).

Furthermore, in ETGP, these indicators increased (number of nodes, 36; coefficient, 0.889; heterogeneity, 0.708; shortest path length, 202; average neighbor number, 5.611; Figure 6c), suggesting that interactions became more complicated with the addition of the enzyme. In general, the interactions within and between the microflora and physicochemical indicators indicated possible microflora competition and supplementation [45]. Thus, the exogenous enzyme additive could change the substrate characteristics and enrich trophic levels to induce more interactions. *Pediococcus, Epicoccum*, and *Alternaria* were the most dominant genera (average RA > 10%) in the co-occurrence network of ETGP. *Pediococcus* was negatively correlated with *Epicoccum*, *Gibberella*, and pH, and positively correlated with *unclassified_k__Fungi*, *Candida*, *Monographella*, fermentation time, CF, CP, and the contents of both lactic acid and ethanol. *Alternaria* was positively related to *Xeromyces* and *Phosphoremia*, and negatively correlated with *Lactobacillus*, *Xerochrysium*, and *Cutaneotrichosporon*. In summary, exogenous probiotics decoupled the interactions among microflora and physicochemical indicators, whereas exogenous enzymes induced more interactions.

## 4. Conclusions

This research proved that the addition of exogenous probiotics could be a feasible strategy to biotransform vegetable waste into fermented feed, and improved the fermentation quality of cabbage leaf FF via changes to the RA of pathogen and physicochemical indicators (CP and odor, particularly). The exogenous probiotics reduced the growth of aerobic, form-biofilms, and pathogenic taxa in the FF, which resulted in an efficient anaerobic fermentation system with facultatively anaerobic, Gram-positive bacterial communities and undefined saprotroph fungal communities. Exogenous probiotics addition enriched *Pediococcus* and *Saccharomyces*, which were the keystone taxa and played an important role in amending the undesirable microbial community.

## Figures and Tables

**Table 1:** Ingredient list of raw materials.

Raw Materials	DM (g kg^−1^)	CF (g kg^−1^ DM)	CP (g kg^−1^ DM)	ADF (g kg^−1^ DM)	NDF (g kg^−1^ DM)
Soybean meal	887.3 ± 1.2	89.4 ± 0.3	446.9 ± 4.8	105.9 ± 5.8	251.0 ± 6.7
Corn flour	877.3 ± 1.9	52.9 ± 3.5	90.5 ± 6.1	11.8 ± 1.3	24.7 ± 2.0
Wheat bran	880.2 ± 1.5	88.2 ± 2.2	195.4 ± 6.1	100.7 ± 1.7	411.4 ± 1.0
Cabbage waste	59.4 ± 0.7	425 ± 2.6	220.0 ± 5.7	176.9 ± 7.8	233.6 ± 4.8

DM: dry matter; CF: crude fat; CP: crude protein; NDF: neutral detergent fiber; ADF: acid detergent fiber.

**Table 2:** The formulations in every bag of the three fermentation treatment groups.

Experimental Group	Formulations	Additive
CTGP	15% Soybean meal + 15% Corn flour + 15% Wheat bran + 55% Cabbage waste	-
PTGP	CTGP	*S. cerevisia* 9.0 × 10^7^ CFU g^−1^ FM *B. subtilis* 6.6 × 10^6^ CFU g^−1^ FM *L. plantarum* 6.6 × 10^6^ CFU g^−1^ FM
ETGP	CTGP	0.5 wt.% FM of exogenous enzyme

CTGP: the control group; PTGP: the probiotics treatment group; ETGP: the enzymes treatment group; “-”: indicates “without additives”; FM: fresh matter.

**Table 3:** Effect of exogenous additives and fermentation time on the chemical composition of fermented feed.

Items	TR	Time (d)					Mean	SEM	*p*-Value		
		0	2	5	10	15			TR	TI	TR × TI
DM (g kg^−1^)	PTGP	376 ± 9.7 ^Aa^	334 ± 4.7 ^Bb^	299 ± 9.3 ^Bbc^	269 ± 7.4 ^Bc^	270 ± 15.2 ^Bc^	303.0	11.72	***	***	***
	CTGP	377 ± 5.2 ^Aa^	375 ± 13.4 ^Aa^	375 ± 9 ^Aa^	373 ± 12.4 ^Aa^	357 ± 14.3 ^Aa^	369.9				
	ETGP	378 ± 14.3 ^Aa^	351 ± 6.7 ^Aba^	346 ± 17.9 ^Aa^	353 ± 6.3 ^Aa^	351 ± 3.4 ^Aa^	356.5				
CP (g kg^−1^ DM)	PTGP	233 ± 12.1 ^Ac^	275 ± 3.1 ^Bb^	301 ± 6.7 ^Aa^	291 ± 7.6 ^Aab^	292 ± 9.8 ^Aab^	275.6	7.34	***	***	***
	CTGP	232 ± 7.2 ^Ac^	245 ± 2.9 ^Babc^	241 ± 6.3 ^Bbc^	260 ± 4.2 ^Ba^	251 ± 7.6 ^Bab^	246.6				
	ETGP	245 ± 7.9 ^Ab^	249 ± 6.3 ^Aab^	260 ± 6.9 ^Bab^	266 ± 3.1 ^Ba^	263 ± 8.3 ^Bab^	256.5				
CF (g kg^−1^ DM)	PTGP	103 ± 2.6 ^Ac^	121 ± 2.3 ^Cc^	148 ± 10.6 ^Bb^	166 ± 10.3 ^Bab^	183.7 ± 6.1 ^Ba^	147.3	8.28	***	***	***
	CTGP	110 ± 8.3 ^Ac^	128 ± 1.7 ^Bbc^	142 ± 9.3 ^Bb^	167 ± 3.6 ^Ba^	181.6 ± 10.9 ^Ba^	143.2				
	ETGP	102 ± 3.8 ^Ac^	186 ± 2.7 ^Ab^	199 ± 9.5 ^Aab^	218 ± 17.8 ^Aa^	208.4 ± 0.8 ^Aab^	180.9				
ADF (g kg^−1^ DM)	PTGP	91.2 ± 4.3 ^Aa^	85.7 ± 4.0 ^Ba^	84.6 ± 0.6 ^Ca^	89.6 ± 2.5 ^Aa^	92.8 ± 3.5 ^Ba^	88.8	6.54	***	***	***
	CTGP	90.9 ± 4.0 ^Ab^	120 ± 4.1 ^Aa^	116.5 ± 0.8 ^Ba^	110 ± 20.2 ^Aab^	95.6 ± 2.4 ^Bab^	106.3				
	ETGP	86.9 ± 2.5 ^Ab^	129 ± 3.7 ^Aa^	136.2 ± 6.1 ^Aa^	123 ± 9.2 ^Aa^	126.2 ± 5.3 ^Aa^	120.1				
NDF (g kg^−1^ DM)	PTGP	211 ± 9.1 ^Ac^	305 ± 9.3 ^Ca^	230 ± 7.2 ^Bbc^	235 ± 2.2 ^Cb^	224 ± 2.5 B^bc^	236.9	11.75	***	***	***
	CTGP	212 ± 5.4 ^Ac^	384 ± 11.1 ^Ba^	372 ± 8.2 ^Aa^	296 ± 2.2 ^Bb^	295 ± 15.1 ^Bb^	314.4				
	ETGP	207 ± 5.6 ^Ab^	350 ± 2.5 ^Aa^	341 ± 14.6 ^Aa^	322 ± 12.8 ^Aa^	326 ± 29.0 ^Aa^	315.9				

^A–C^: means in the same column followed by different uppercase letters differ (*p* < 0.05); ^a–c^: means in the same row followed by different lowercase letters differ (*p* < 0.05). TR: treatment; TI: time. PTGP: the probiotics treatment group; CTGP: the control group; ETGP: the enzymes treatment group. DM: dry matter content; CP: crude protein; CF: crude fat; NDF: neutral detergent fiber; ADF: acid detergent fiber. The values are shown as the mean ± standard deviation of three replicates. SEM: standard error of means. *** *p* < 0.001.

**Table 4:** Effect of exogenous additives and fermentation time on fermentation characteristics.

Items	TR	Time (d)					Mean	SEM	*p*-Value		
		0	2	5	10	15			TR	TI	TR × TI
pH	PTGP	6.20 ± 0.11 ^Aa^	5.00 ± 0.05 ^Ab^	4.44 ± 0.14 ^Ac^	4.32 ± 0.02 ^Ac^	4.34 ± 0.14 ^Ac^	4.76	0.07	***	***	***
	CTGP	6.22 ± 0.05 ^Aa^	3.96 ± 0.07 ^Bb^	3.89 ± 0.01 ^Bb^	3.89 ± 0.04 ^Bb^	3.88 ± 0.02 ^Bb^	4.23				
	ETGP	6.14 ± 0.12 ^Aa^	4.07 ± 0.01 ^Bb^	3.93 ± 0.02 ^Bbc^	3.92 ± 0.02 ^Bc^	3.96 ± 0.01 ^Bc^	4.28				
Lactic acid (g kg^−1^ DM)	PTGP	0.0 ± 0.0 ^Cc^	36.2 ± 2.7 ^Bb^	63.4 ± 11.9 ^Aa^	52.7 ± 8.1 ^Bab^	39.9 ± 2.9 ^Bb^	36.4	4.43	***	***	***
	CTGP	2.5 ± 0.1 ^Bc^	75.5 ± 3.3 ^Ab^	79.6 ± 6.3 ^Aab^	82.2 ± 4.3 ^Aab^	88.0 ± 2.6 ^Aa^	64.0				
	ETGP	4.1 ± 0.2 ^Ac^	68.9 ± 3.3 ^Ab^	83.0 ± 3.8 ^Aa^	87.1 ± 4.2 ^Aa^	89.7 ± 1.2 ^Aa^	66.5				
Acetic acid (g kg^−1^ DM)	PTGP	0.0 ± 0.0 ^Cc^	10.7 ± 0.8 ^Aab^	13.4 ± 4.3 ^Aa^	8.8 ± 1.5 ^Aab^	5.5 ± 1.6 ^Bb^	7.3	1.14	***	***	***
	CTGP	0.5 ± 0.0 ^Bb^	5.6 ± 0.7 ^Ba^	4.8 ± 0.9 ^Ba^	5.2 ± 0.3 ^Ba^	5.3 ± 0.9 ^Ba^	4.3				
	ETGP	0.3 ± 0.1 ^Ac^	9.2 ± 0.5 ^Ab^	11.3 ± 0.8 ^ABa^	10.2 ± 0.6 ^Aab^	9.6 ± 0.6 ^Ab^	7.9				
Butyric acid (g kg^−1^ DM)	PTGP	0.0 ± 0.0 ^Ab^	0.9 ± 0.0 ^Aab^	1.7 ± 0.9 ^Aa^	0.7 ± 0.0 ^Aab^	0.5 ± 0.0 ^Aab^	0.7	0.21	***	***	***
	CTGP	0.0 ± 0.0 ^Ab^	0.4 ± 0.2 ^Bab^	0.4 ± 0.0 ^Aa^	0.6 ± 0.1 ^Aa^	0.7 ± 0.2 ^Aa^	0.4				
	ETGP	0.0 ± 0.0 ^Ad^	0.5 ± 0.1 ^ABc^	0.7 ± 0.1 ^Ab^	0.7 ± 0.0 ^Ab^	0.9 ± 0.0 ^Aa^	0.5				
Ethanol (g kg^−1^ DM)	PTGP	0.0 ± 0.0 ^Ab^	55.5 ± 4.7 ^Ba^	66.3 ± 19 ^Ba^	62.7 ± 10.3 ^Ba^	39.2 ± 1.4 ^Ca^	41.9	5.08	***	***	***
	CTGP	0.0 ± 0.0 ^Ac^	1.9 ± 0.1 ^Bb^	1.9 ± 0.2 ^Bb^	21.5 ± 1.2 ^Ba^	20.3 ± 0.0 ^Ba^	7.8				
	ETGP	0.0 ± 0.0 ^Ac^	1.4 ± 0.1 ^Abc^	2.9 ± 0.5 ^Ab^	19.1 ± 0.9 ^Aa^	1.6 ± 0.0 ^Ab^	4.5				
NH_4_^+^/TN (%)	PTGP	0.46 ± 0.02 ^Bc^	1.44 ± 0.21 ^Bb^	2.21 ± 0.39 ^Aab^ 2.45 ± 0.40 ^Ba^	1.90 ± 0.34 ^Bab^	1.90 ± 0.34 ^Bab^	1.78	0.23	***	***	***
	CTGP	0.79 ± 0.06 ^Ae^	1.36 ± 0.15 ^Bd^	2.30 ± 0.02 ^Ab^	3.27 ± 0.17 ^Aa^	1.87 ± 0.06 ^Bc^	2.00				
	ETGP	0.53 ± 0.02 ^Bd^	2.01 ± 0.10 ^Ac^	2.39 ± 0.19 ^Abc^ 2.68 ± 0.34 ^ABb^	3.52 ± 0.14 ^Aa^	3.52 ± 0.14 ^Aa^	2.26				

^A–C:^ means in the same column followed by different uppercase letters differ (*p* < 0.05); ^a–c^: means in the same row followed by different lowercase letters differ (*p* < 0.05). TR: treatment; TI: time. PTGP: the probiotics treatment group; CTGP: the control group; ETGP: the enzymes treatment group. DM: dry matter content; The values are shown as the mean ± standard deviation of three replicates. SEM: standard error of means. *** *p* < 0.001.

**Table 5:** Fermented quality of fermented feed evaluated by Flieg’s score based on the concentration of organic acid.

Item	TR	Time (d)					Mean	SEM	*p*-Value		
		0	2	5	10	15			TR	TI	TR × TI
Formic acid (g kg^−1^ DM)	PTGP	7.99 ± 0.29 ^Ac^	34.01 ± 2.1 ^Aa^	30.44 ± 3.69 ^Aa^	31.47 ± 1.73 ^Aa^	20.2 ± 2.96 ^ABb^	24.19	1.48	***	***	***
	CTGP	8.84 ± 0.44 ^Ab^	9.72 ± 1.22 ^Bb^	11.31 ± 1.01 ^Bb^	23.74 ± 0.54 ^Ba^	22.79 ± 1.78 ^Aa^	14.05				
	ETGP	6.76 ± 0.29 ^Bc^	10.01 ± 0.82 ^Bb^	13.23 ± 1.41 ^Ba^	15.66 ± 0.98 ^Ca^	13.48 ± 0.08 ^Ba^	11.59				
Propionic acid (g kg^−1^ DM)	PTGP	NA	NA	NA	NA	NA	-	-	-	-	-
	CTGP	NA	NA	NA	NA	NA	-				
	ETGP	NA	NA	NA	NA	NA	-				
Lactic acid/TOA (%)	PTGP	-	44.25	58.20	56.26	60.37	-	-	-	-	-
	CTGP	-	82.77	82.83	73.56	75.35	-				
	ETGP	-	77.76	76.69	76.63	78.90	-				
Acetic acid/TOA (%)	PTGP	-	13.08	12.30	9.39	8.32	-	-	-	-	-
	CTGP	-	6.14	4.99	4.65	4.54	-				
	ETGP	-	10.38	10.44	8.97	8.44	-				
Butyric acid/TOA (%)	PTGP	-	1.10	1.56	0.75	0.76	-	-	-	-	-
	CTGP	-	0.44	0.42	0.54	0.60	-				
	ETGP	-	0.56	0.65	0.62	0.79					
Flieg’s score	PTGP	-	Good (80)	Very good (87)	Very good (86)	Very good (88)	-	-	-	-	-
	CTGP	-	Very good (100)	Very good (100)	Very good (97)	Very good (100)	-				
	ETGP	-	Very good (100)	Very good (100)	Very good (100)	Very good (100)	-				

DM: dry matter; TOA: total organic acid including the content of lactic acid, formic acid, acetic acid, propionic acid, butyric acid. PTGP: the probiotics treatment group; CTGP: the control group; ETGP: the enzymes-treatment group. “-”: not calculated; “NA”: not detected. TR: treatment; TI: time. ^A–C^: means in the same column followed by different uppercase letters differ (*p* < 0.05); ^a–c^: means in the same row followed by different lowercase letters differ (*p* < 0.05). The values are shown as the mean ± standard deviation of three replicates. SEM: standard error of means. *** *p* < 0.001.

## Data Availability

The datasets generated for this study can be found in Sequence Read Archive under BioProject, PRJNA642655 (http://www.ncbi.nlm.nih.gov/sra/, accessed on 17 March 2021).

## Supplementary Materials

The following are available online at https://www.mdpi.com/2076-2607/9/3/644/s1, Table S1: Effect of exogenous additives and fermentation time on α diversity indicators of fermented feed.
