# Supplementary material for: Exogenous Probiotics Improve Fermentation Quality, Microflora Phenotypes, and Trophic Modes of Fermented Vegetable Waste for Animal Feed
Source: Microorganisms. 2021 Mar 19;9(3):644. doi: 10.3390/microorganisms9030644 (PMC8003719; doi:10.3390/microorganisms9030644)
Supplement: Supplementary file 1 [file microorganisms-09-00644-s001.zip › Table S1.pdf]

Table S1 Effect of exogenous additives and fermentation time on α diversity indicators of fermented feed.

α index	Group	Day 0	Day 2	Day 5	Day 10
Shanon	PTGP	2.12[a] / 1.99[b]	0.05 / 0.06	0.24 / 0.09	0.15 / 0.09
	CTGP	1.57 / 2.14	0.66 / 2.87	0.55 / 2.59	0.81 / 2.65
	ETGP	1.93 / 2.19	1.21 / 3.04	0.97 / 2.81	0.91 / 3.17
Chao1	PTGP	131.05 / 264.20	10.00 / 49.14	24.50 / 56.13	17.50 / 50.00
	CTGP	85.40 / 207.48	20.00 / 119.50	14.60 / 148.25	17.67 / 89.25
	ETGP	92.83 / 165.77	13.50 / 118.00	30.00 / 96.20	15.00 / 86.50
Coverage	PTGP	0.978 / 0.998	0.998 / 1.000	0.996 / 1.000	0.997 / 1.000
	CTGP	0.986 / 0.999	0.997 / 1.000	0.998 / 1.000	0.997 / 1.000
	ETGP	0.990 / 0.999	0.999 / 1.000	0.997 / 1.000	0.998 / 1.000

PTGP: the probiotics-treatment group; CTGP: the control group; ETGP: the enzymes-treatment group.

[a,b]:indicate the α index for bacterial community and fungal, respectively. Shannon, Chao1, and Coverage indicators indicate diversity, richness, and sequencing accuracy
