# Supplementary material for: Exogenous Probiotics Improve Fermentation Quality, Microflora Phenotypes, and Trophic Modes of Fermented Vegetable Waste for Animal Feed
Source: Microorganisms. 2021 Mar 19;9(3):644. doi: 10.3390/microorganisms9030644 (PMC8003719; doi:10.3390/microorganisms9030644)
Supplement: Supplementary file 1 [file microorganisms-09-00644-s001.zip › Figure S1.pdf]

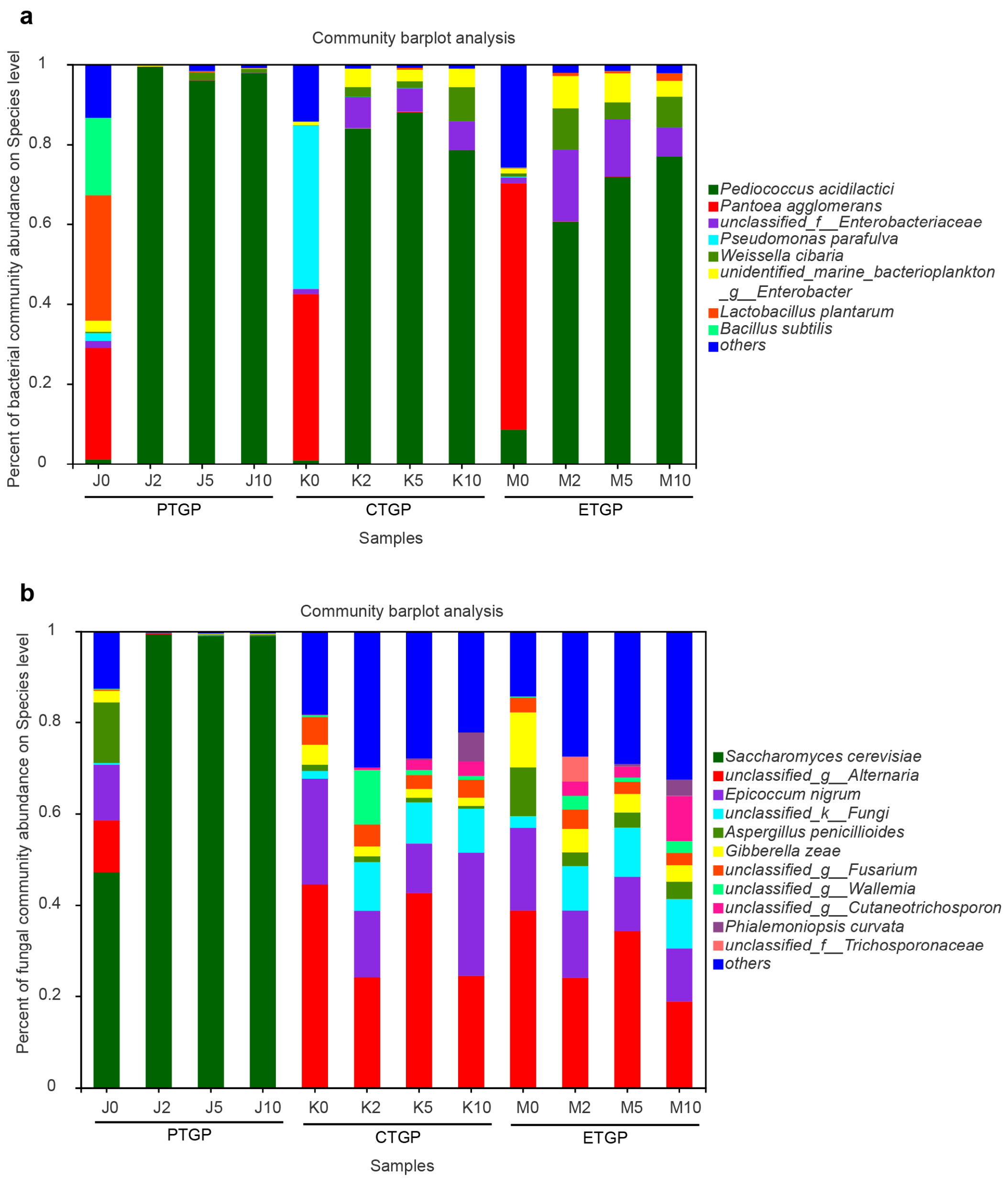

Figure S1. The relative abundance of microbial community on species level in vegetable byproduct fermented feed.

PTGP: the probiotics-treatment group; CTGP: the control group; ETGP: the enzymes-treatment group.
